# Effects of *Lactobacillus fermentum* Administration on Intestinal Morphometry and Antibody Serum Levels in *Salmonella*-Infantis-Challenged Chickens

**DOI:** 10.3390/microorganisms11020256

**Published:** 2023-01-19

**Authors:** Miroslava Anna Šefcová, David Ortega-Paredes, César Marcelo Larrea-Álvarez, Iván Mina, Victoria Guapás, David Ayala-Velasteguí, Paula Leoro-Garzón, Gabriel Molina-Cuasapaz, Christian Vinueza-Burgos, Viera Revajová, Marco Larrea-Álvarez

**Affiliations:** 1 Facultad de Ciencias Médicas Enrique Ortega Moreira, Carrera de Medicina, Universidad Espíritu Santo, Samborondón 092301, Ecuador; 2 Unidad de Investigación en Enfermedades Transmitidas por Alimentos y Resistencia a los Antimicrobianos (UNIETAR), Facultad de Medicina Veterinaria y Zootecnia, Universidad Central del Ecuador, Quito 170129, Ecuador; 3 Research Unit, Life Science Initiative (LSI), Quito 170102, Ecuador; 4 School of Biological Science and Engineering, Yachay-Tech University, Urcuquí 100650, Ecuador; 5 Biomedical Research Unit, Inmunolab, Quito 170136, Ecuador; 6 Facultad de Ciencias Agropecuarias y Recursos Naturales, Carrera de Medicina Veterinaria, Universidad Técnica de Cotopaxi, Latacunga 050101, Ecuador; 7 Department of Morphological Disciplines, University of Veterinary Medicine and Pharmacy, 040 01 Košice, Slovakia

**Keywords:** *Lactobacillus fermentum*, *Salmonella enterica* subsp. *enterica* serovar Infantis, broiler chickens, small intestine, villus height, crypt depth, surface, goblet cell count, *muc-2* expression levels, IgM serum levels

## Abstract

There are no studies reporting the effects of *Salmonella enterica* subsp. *enterica* serovar Infantis (*S*. Infantis) on intestinal architecture and immunoglobulin serum levels in chickens. Here, we measured these parameters and hypothesized whether probiotic administration could modulate the observed outcomes. Two-hundred 1-day-old COBB 500 male chicks were allocated into four groups: (I) the control, (II) the group treated with *L. fermentum*, (III) the group exposed to *S*. Infantis, and (IV) the group inoculated with both bacteria. At 11 days post infection, blood was gathered from animals which were then euthanized, and samples from the small intestine were collected. Intestinal conditions, as well as IgA and IgM serum levels, were assessed. *S*. Infantis reduced villus-height-to-crypt-depth (VH:CD) ratios in duodenal, jejunal, and ileal sections compared to control conditions, although no differences were found regarding the number of goblet cells, *muc-2* expression, and immunoglobulin concentration. *L. fermentum* improved intestinal measurements compared to the control; this effect was also evidenced in birds infected with *S*. Infantis. IgM serum levels augmented in response to the probiotic in infected animals. Certainly, the application of *L. fermentum* elicited positive outcomes in *S*. Infantis-challenged chickens and thus must be considered for developing novel treatments designed to reduce unwanted infections.

## 1. Introduction

*Salmonella enterica* has emerged across the globe as a threat to health systems [1]. Serovars of this species can infect humans, causing diverse effects varying from typhoid fever to gastroenteritis [2]. Non-typhoidal *Salmonella* (NTS) is associated with millions of infections and thousands of deaths annually around the globe [3,4]. *S*. Typhimurium and *S*. Enteritidis are considered the principal serovars associated with human infections; nevertheless, *S. enterica* subsp. *enterica* serovar Infantis (*S*. Infantis) has emerged as a relevant serovar causing salmonellosis in humans [5]. Since *S*. Infantis has been reported as a dominant serovar isolated from poultry and human sources [6,7], it should be considered a global emerging threat to public health. Furthermore, various isolates have shown resistance to multiple drugs and enhanced pathogenicity, which has been linked to the acquisition of a virulence-resistant plasmid known as the plasmid of the emerging *S*. Infantis (pESI) that encodes virulence factors and the antibiotic and mercury resistance genes [8]. Antibiotic resistance genes have also been associated with integrons, which may contribute to their mobility among *S*. Infantis strains [9].

Poultry, as well as pigs, are considered the main reservoirs [10]. Thus, various studies have focused on testing different approaches for decreasing *S*. Infantis colonization, including algae and probiotics. For instance, inoculation with probiotic strains have reduced *S*. Infantis levels in the guts of pigs and broiler chickens [11,12], which is associated with the capacity of these bacteria to produce short-chain fatty acids, secrete antimicrobials, stimulate the immune system, or competitively exclude other bacteria [13]. On the other hand, dietary administration of the green microalga *Tetraselmis chuii* did not alter *S*. Infantis cecal load in broilers, despite the presence of fermentable polysaccharides as part of the cell wall [14]. Such polysaccharides are known for modulating cecal microbiota [15], and it has been shown that application of seaweed-derived polysaccharides reduced *S*. Enteritidis levels in laying hens [16].

Serovars of *Salmonella*, such as *S.* Pullorum and *S.* Typhimurium, are known for causing mucosal damage of the small intestine [17,18]. As *S*. Infantis is not considered a significantly invasive serovar [19], information concerning its effects on intestinal architecture in broiler chickens is not available despite the importance of the gut epithelium as a barrier against invading pathogens, in nutrient acquisition, and in host immunity [20,21]. *S.* Pullorum, for instance, is known for eliciting production of both IgA and IgM [22]. However, immunoglobulin serum levels, in response to a *S*. Infantis infection, have not been reported so far. In this study, we not only aimed to measure such parameters but also to determine whether inoculation with a probiotic strain could modulate the observed effects. Indeed, probiotic strains, including *Bacillus subtilis* and *Lactobacillus acidophilus*, have relieved the intestinal damage initiated by *S*. Enteritidis and *S*. Typhimurium in broiler chickens [23]. Administration of *L. fermentum* has proved useful not only for enhancing the immune reaction of broiler chickens challenged with *Campylobacter coli* and *C. jejuni* but also for diminishing the intestinal damage induced by the latter [24,25,26]. *Lactobacillus* species harbor associated molecular patterns that are capable of activating NLRP (Nod-like receptor protein) and Toll-like receptors in epithelial and dendritic cells, triggering differential cytokine expression that promotes enterocyte differentiation via cellular signaling or cytokine secretion, including IL-22, IL-1β, IL-13, and IL-4 [27]. Similarly, recognition of these molecular patterns, by the aforementioned receptors, induces polarization of helper cells and the concomitant synthesis of interleukins that prompt production of antibodies by activated B cells [28]. 

Reports documenting the effects of *S*. Infantis on intestinal architecture and immunoglobulin serum levels in broiler chickens are not available. Thus, this study aimed to examine such parameters. Moreover, we hypothesized whether probiotic administration could influence the measured outcomes. Consequently, we treated animals with *L. fermentum* and infected them with *S*. Infantis. Histological measurements were used to determine intestinal architecture; transcript abundance of *muc-2* and serum antibody levels were also assessed using reverse-transcriptase quantitative PCR (RT-qPCR) and ELISA, respectively.

## 2. Materials and Methods

### 2.1. Ethics Statement

All experimental procedures were performed following the guidelines for animal management specified by the Agency for the Regulation and Control of Phytosanitary and Animal Health (AGROCALIDAD, technical resolution n. 0017). The study was approved by the Ethics Committee on the Use of Animals in Research and Teaching of the San Francisco de Quito University (USFQ) (reference number: 2020-008).

### 2.2. Experimental Design, Housing Conditions, and Animal Management

The experimental Center for Animal Research of the Veterinary Medicine Faculty, Universidad Central del Ecuador, was used to carry out the experiments. A total of two-hundred 1-day-old COBB 500 broiler male chicks were subjected to experimental conditions. Chickens were assigned at random into four experimental groups: (I) the control group, in which a saline solution (0.2 mL) was applied individually to birds each time their counterparts were inoculated; (II) the *L. fermentum* group, in which birds were treated with a suspension of the probiotic (10^9^ colony-forming units [CFU]/0.2 mL) that was applied from days 1 to day 7 of the experimental period [26]; (III) the *S*. Infantis group, infected with a suspension of the bacteria (10^7^ CFU/0.1 mL) on day 4 [14,19]; and finally, in group IV, animals were exposed to the probiotic during the first week and inoculated with *S*. Infantis on the fourth day. The experiment lasted 15 days. 

Bacterial strains were administered orally (Table S1); they were prepared as described previously [14,26]. The *L. fermentum* strain CCM7514 was provided in a lyophilized form by the Czech Collection of Microorganisms (CCM), Brno, Czechia; the strain originated from the intestine of domestic chickens. Saline solution (1 mL) was used for bacterial resuspension; *L. fermentum* growth was carried out inside an anaerostate (BBL GasPak Plus, Albany, NY, USA) at 37 °C for 48 h using De Mann–Rogosa–Sharpe (MRS) agar (Merck, Germany). Solitary colonies, at least five, were inoculated in MRS broth (50 mL) and incubation was performed for 24 h at 37 °C. After cultivation, MRS broth was added (450 mL) and centrifugation took place for 45 min at 2268× *g* at 4 °C. Resuspension of the resulting sediment was carried out with saline solution (50 mL); decimal dilutions were performed to assess the number of bacteria. Each animal was inoculated *per os* with 10^9^ colony-forming units [CFU]/0.2 mL from the first to the seventh day of the experiment [26]. *S.* Infantis growth was carried out using pure cultures (1 × 10^9^ CFU/mL); bacterial recovery was achieved on differential selective medium (XLD, Xylose, Lysine, and Deoxycholate) at 37 °C for 24 h. For biomass generation, characteristic colonies were selected for liquid cultures (buffered-peptone water), which were later incubated at 37 °C with constant agitation for 18 to 24 h. The generated biomass was collected in tubes and centrifuged at 500× *g* for 45 min for biomass concentration. Pellet resuspension was carried out with saline solution (NaCl 5%) until reaching an OD_600_ of around 1.0. The solution was arranged in series using plate count agar, and adjusted at around 1–2 × 10^7^ CFU/0.1 mL. Each bird was inoculated orally with 10^7^ CFU/0.1 mL on the fourth day of the experiment [14].

Experimental groups were allocated in individual pens of 3 m × 3 m of 50 chickens each. The animal was considered the experimental unit (EU), since they were independently allocated to treatment conditions and experimental interventions; moreover, each EU could not influence each other on the measured outcomes [29]. Animals were provided a commercial feed, without coccidiostats, antibiotics, or probiotics, for starter (0–8 days) and grower (9–14 days) diets (Supplementary Materials, Table S2) [30]; they had access to feed and water ad libitum during the entire experimental period. Relative humidity was maintained between 50–70%. During the first day of placement, a regime of continuous light was provided (intensity 30–40 Lux). From day 2, light was turned off for 1 h until the birds reached 130–180 g, then a regime of 18 h of light (intensity 5–10 Lux) and 6 h of dark was provided until the end of the experiment (day 15). Hardwood shavings were used to cover the floor where birds were raised. During the first week, temperature was kept between 30 and 32 °C; it was decreased by 2 °C per week, on day 7 (28–30 °C) and on day 14 (25–27 °C). Housing and management abided by the COBB 500 Management Guide [31]. On day 15 (11 dpi, days post infection), 10 birds (*n* = 10) were selected per experimental group and blood samples were collected from the brachial vein. Then, animals were electrically stunned and finally euthanized by bleeding.

### 2.3. Histological Analyses

From the intestine, the loop of the duodenum; the mid-point of the jejunum, located between the point of entry of the bile duct and Meckel’s diverticulum; along with the mid-point of the ileum, located between Meckel’s diverticulum and the ileocecal junction, were collected (2 cm of each segment) [24]. A solution of formalin (10%) was utilized to fix the samples for 2 days, and they were then serially washed with ethyl alcohol (70%, 90% and 100%) for dehydration. Xylol was used to diaphanize the samples, which were later embedded in blocks of paraffin. A rotary microtome (Leica RM2235, Wetzlar and Mannheim, Germany) was employed to slice the blocks in three longitudinal sections of 5 μm; staining was carried out using hematoxylin and eosin (HE staining). The Motic Images Plus 2.0 software (Motic, Hong Kong, China) was utilized for capturing and processing images from intestinal sections (duodenum, jejunum, and ileum). Villus height, villus width, and crypt depth were assessed in each of these segments. At least six uninjured villi were selected and the procedure was performed 4 times for a total of 24 readings per chicken. An intact lamina propria was used as a reference for villus choice. Surface area was calculated using the following formula: [2π × (villus width/2) × villus height] as described by [32]. The villus-height-to-crypt-depth ratio was estimated as detailed previously [24]. The Motic Images Plus 2.0 software was used to assess the number of goblet cells in 10 intact villi, and this was estimated per 100 intestinal epithelial cells [33].

### 2.4. RNA Extraction, Reverse Transcription, and Quantitative Polymerase Chain Reaction Assays

A section of the ileum was kept in RNA later and stored at −80 °C. The tissue was thawed and homogenized by manual grinding for approximatively 10 min using 1 mL of TRIzol™ reagent (Thermo Scientific, Waltham, MA, USA). After extraction, samples were left at −20 °C for 10 min. Subsequently, 4-bromoanisole (Sigma-Aldrich Inc., St. Louis, MO, USA) (50 µL) was added to the tubes, which were later shaken. Centrifugation of the mix was performed at 12,000× *g* for 15 min. The extracted RNA was precipitated and purified using the AccuPrep Universal RNA Extraction Kit (BioNeer Corporation, Daejeon, Republic of Korea) according to the provided guidelines. RNA quality and concentration were assessed with a NanoDrop One spectrophotometer (Thermo Scientific, Waltham, MA, USA). The RNA samples were stored at −80 °C.

Reverse transcription was performed with the OneScript Plus cDNA Synthesis Kit (Applied Biological Materials Inc., Vancouver, Canada). The components were thawed and mixed before use; reactions were performed on ice. The extracted RNA was mixed with buffer, dNTPs, primers, nuclease-free water, and the OneScript RTase^®^. Synthesis of cDNA was carried out by incubating for 15 min at 50–55 °C. For removal of complementary RNA, 1 µL of *E*. *coli* RNase H (Applied Biological Materials Inc., Vancouver, Canada) was added, followed by incubation for 20 min at 37 °C. Dilution of the cDNA was carried out in 10× in UltraPure™ DNase/RNase-Free distilled water (Invitrogen, Waltham, MA, USA) and kept at −80 °C. For quantitative analysis, the following primers were utilized: *muc-2* Forward 5′-GCCTGCCCAGGAAATCAAG-3′ and Reverse 5′-CGACAAGTTTGCTGGCACAT-3′ [34]. GAPDH was used as a housekeeping gene, primers were as follows: Forward 5′-CCTGCATCTGCCCATTT-3′ and Reverse 5′-GGCACGCCATCACTATC-3′ [35]. Cycling conditions, detection, amplification, calculation of melting curve, and data normalization were set as described previously [36]. The primers used for analyses allowed amplification efficiencies between 94% and 100%. The Stratagene Mx3000P Multiplex QPCR (Agilent, Sta. Clara, CA, USA) was used for amplification and detection of specific sequences. The cycling conditions were as follows: initial denaturation for 5 min at 95 °C, which was followed by 36 cycles at 95 °C for 20 s. The annealing step was performed at 57 °C for 30 s and the extension step at 72 °C for 30 s. A melting curve ranging from 50 °C to 95 °C, with readings every 0.5 °C, was carried out for each RT-qPCR plate. Samples were evaluated in duplicate and means were used for calculations. The reference gene (GAPDH) was employed to normalize Ct values (Delta—Δ—Ct) that were calculated as 2^−Δ^Ct [37].

### 2.5. Antibody Determination

Blood was collected from the brachial vein with 4 mL vacuum tubes; this procedure was restricted to a maximum of 2 min. Samples were kept at room temperature for 120 min and stored at 4 °C overnight. Centrifugation was then carried out at 2500× *g* at 4 °C for 10 min, and the serum was maintained at −80 °C. Detection of IgA and IgM levels was carried out by an enzyme-linked immunosorbent assay (ELISA) using chicken IgA and IgM ELISA kits (Abcam, Cambridge, UK), following the provided instructions. Briefly, 100 µL of IgA or IgM standards, along with diluted serum samples and suitable controls were added to selected wells in duplicates. The IgA plate was incubated at room temperature for 20 min and the IgM plate for 30 min, which was followed by treatment with a wash buffer (4 times). Then, 100 µL of anti-chicken IgA or IgM—HRP (horseradish peroxidase) conjugate antibody were added to each well and incubated at room temperature in the dark for 20 (IgA) and 30 min (IgM). After the washing steps (4 times), 100 µL of the chromogen solution 3,3,5,5′-tetramethylbenzidine (TMB) was added to each well. Following incubation at room temperature (10 min), the reaction was halted with 100 µL of stop solution. Absorbance was measured at 450 nm in a Multiskan EX microplate reader (Thermo Scientific, Waltham, MA, USA). Reads were carried out in duplicates, which were averaged for further analyses. Control values were subtracted from treatment values. Antibody concentration was determined using a standard curve generated with the GraphPad Prism 9 Software (San Diego, CA, USA).

### 2.6. Statistical Analysis

Analyses were performed in MATLAB version 9.9.9341360 (MathWorks, Natick, MA, USA) (R2016a). Normality was assessed using the Shapiro–Wilk’s test, and homogeneity of variance was calculated with Levene’s test. A one-way analysis of variance and a Tukey post hoc test were utilized to determine differences between groups when data was homoscedastic and normally distributed. For normally distributed and heteroscedastic data, Welch’s ANOVA and Welch’s t-test were applied. The Kruskal–Wallis test and the Mann–Whitney U test (Wilcoxon rank sum test) were used when data were non-normally distributed. In this case, medians were used as a measure of the tendency of distribution as means are affected due to the non-symmetrical distribution.

## 3. Results

### 3.1. Intestinal Parameters

**Figure 1:**
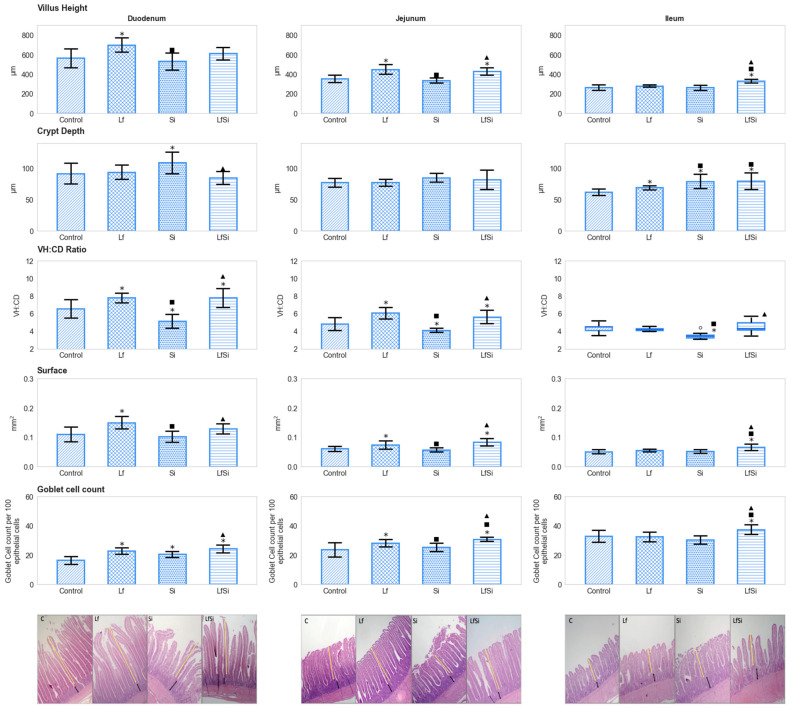
Effects of bacterial administration on morphological characteristics of the small intestine in broiler chickens with respective illustrative photomicrographs (40× magnification, HE staining). Values are means plus SD (*n* = 10). Box plots are used for depicting medians with their corresponding interquartile range (IQR). * designates differences from the control group (*p* < 0.05), ■ from the Lf group, and ▲ from the Si group. Lf: *L. fermentum*; Si: *S*. Infantis; VH: villus height; CD: crypt depth; SD: standard deviation. Circles denote outliers. Yellow and black double-headed arrows indicate villus height and crypt depth, respectively.

In all sections, exposure to *S*. Infantis did not influence the height of villi compared to the control. Probiotic administration, on the other hand, proved to increase this condition in both the duodenum and jejunum; chickens exposed to *L. fermentum* showed taller villi than control and *S*. Infantis-infected birds (*p* < 0.05). In jejunal sections, the positive effect of *L. fermentum* was observed even in the presence of *S*. Infantis. Exposure to both microorganisms augmented the height of villi in ileal sections (*p* < 0.05), although no effects were observed when bacteria were administered individually (*p* > 0.05) (Figure 1) (Table S3). In duodenal and ileal sections, inoculation with *S*. Infantis resulted in deeper crypts compared to control conditions. In the duodenum, such an arrangement was not detected in the presence of the probiotic. In animals exposed to all experimental conditions, ileal sections showed deeper crypts than those of control animals (*p* < 0.05) (Figure 1). A higher villus-height-to-crypt-depth ratio was determined in the duodenum and jejunum of chickens exposed to the probiotic than in those of the control group. On the other hand, infection with *S*. Infantis reduced such values in all sections. This negative effect was not observed when infected birds were previously treated with *L. fermentum* (*p* < 0.05) (Figure 1). In the duodenum and jejunum, animals of the *L. fermentum* group showed larger surface areas than those of the control and *S*. Infantis group. In jejunal sections, this improvement was observed even in the presence of *S*. Infantis. In ileal sections, simultaneous exposure to both bacteria yielded higher surface values in treated chickens than in control conditions (*p* < 0.05) (Figure 1). Similarly, the number of goblet cells in the duodenum, jejunum, and ileum was augmented in animals treated with both bacteria compared to those of the control. In duodenal and jejunal sections, higher values were registered in the probiotic than in the control group (*p* < 0.05) (Figure 1). Administration of the probiotic as well as infection with *S*. Infantis did not modify transcript abundance of *muc-2* (*p* > 0.05) (Table 1) (Table S3).

### 3.2. Antibody Serum Levels

**Figure 2:**
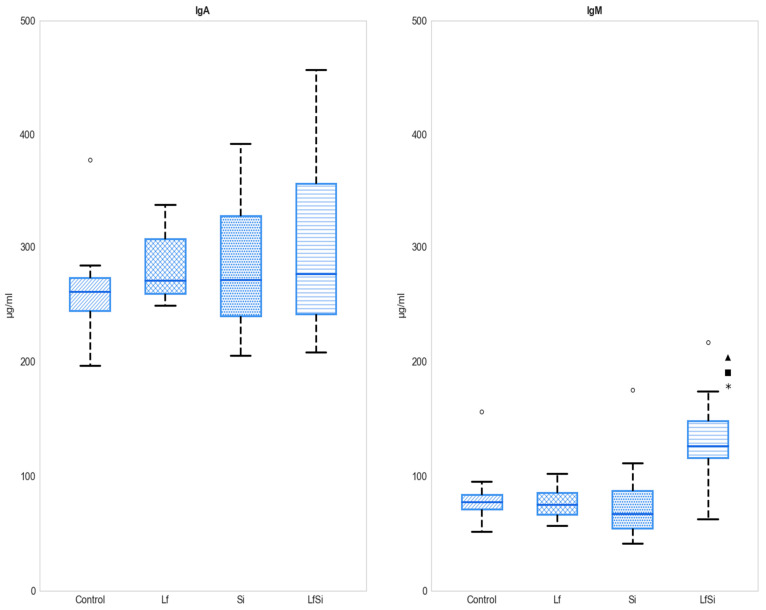
Effects of bacterial treatments on IgA and IgM serum levels (*n* = 10). Box plots are used for depicting medians with their corresponding interquartile range (IQR). * designates differences from the control group (*p* < 0.05), ■ from the Lf group, and ▲ from the Si group. Lf: *L. fermentum*; Si: *S*. Infantis. Circles denote outliers.

No differences in IgA serum levels were found between experimental groups. IgM concentration was only altered when animals were inoculated with both *L. fermentum* and *S*. Infantis. Levels detected in the co-exposure group were higher than those detected in the other groups (Figure 2) (Table S3).

## 4. Discussion

*S*. Infantis has emerged as a significant serovar commonly reported in poultry products [38,39,40], and its spreading could be considered of importance for public health [41,42]. Various studies have focused on testing different approaches for successfully decreasing *S*. Infantis cecal colonization not only in broiler chickens but also in pigs [11,12]. However, there is no information regarding its effects on intestinal architecture and immunoglobulin serum levels in broiler chickens, since it is not considered as invasive as other serovars [19]. Besides measuring such effects, this investigation sought to determine the influence of *L. fermentum* administration during a *S*. Infantis infection. Probiotic treatment not only relieved the intestinal effects elicited by *S*. Infantis but also improved levels of serum IgM in 15-day-old chickens. This study represents the earliest report demonstrating that *L. fermentum* can play a protective role in the intestines of birds infected with the aforementioned serovar.

Intestinal epithelial cells are considered crucial constituents of the gut ecosystem as they not only partake in protection against invading pathogens but are also involved in nutrient acquisition [43,44]. Alternatives to improve intestinal architecture have been tested with success, including plant extracts, microalgae biomass, or probiotics [24,45,46]. *Salmonella* serovars, including *S*. Pullorum and *S*. Typhimurium, are known for eliciting mucosal damage of the small intestine [17,18]. Probiotic bacteria, on the other hand, help preserve the integrity of the epithelium [47,48,49], which stimulates the absorption of nutrients and ultimately leads to a superior growth performance [50,51]. *L. fermentum*, as many other *Lactobacillus* species, has proved useful for ameliorating intestinal health of broiler chickens [24,52,53,54]. Indeed, this probiotic species improved the height of villi in duodenal and jejunal sections, but it did not influence crypt depth. *S*. Infantis did not alter villus height, although it led to the development of deeper crypts in the duodenum and ileum. Longer villi are associated with improved nutrient intake due to an increase of absorptive surface; indeed, shortening of the intestinal villi has been linked to poor gut health [55,56]. Deeper crypts have been related to an active regeneration of the villi [57], although an increase in crypt depth, associated with a decrease in villus height, might result in an augmented metabolic cost of epithelium turnover [58]. Crypt stem cells divide, differentiate, and migrate upwards, providing cells for villus development. Shallow crypts indicate a greater number of mature cells, thus improving feed utilization [17].

The villus-height-to-crypt-depth ratio is an effective parameter for assessing intestinal integrity; when this ratio increases, it is assumed that digestion and absorption are ameliorated [59]. The assessed values were larger in birds treated with the probiotic than in control and *Salmonella*-infected animals. Birds from the latter group showed even lower ratios than those of untreated chickens. The improvement observed with regard to intestinal architecture could help ameliorate the capacity for digestion and absorption, as suggested by the increase of villi absorptive area observed in animals exposed to *L. fermentum*. Microbial synthesis of fermented products (e.g., short-chain fatty acids) modulate intestinal epithelium proliferation [60], and exposure to lactic acid bacteria has proved to accelerate the crypt–villus axis movement of intestinal enterocytes by activating integrin collagen receptors [61]. A large area of villi is capable of rapidly absorbing nutrients from digested food; thus, a loss of this absorptive surface may inhibit nutrient intake, leading to alimentary deficiency and even intestinal failure [62]. It has been reported that infection by *S*. Typhimurium and *S*. Enteritidis reduced intestinal surface area in broilers [63,64]. *S*. Infantis did not modify this parameter in comparison to control animals, although measured values were lower than those found in probiotic-treated chickens. In birds colonized by *L. fermentum* with prior infection with *S*. Infantis, villi surface area was larger than that of only infected chickens, and in the jejunum and ileum, it was larger than that of control animals. Clearly, the use of the probiotic triggered beneficial effects in the presence or absence of *S*. Infantis. Similar results have been hitherto reported in the context of infections associated with other serovars. For instance, addition of *B. subtilis* increased villus surface area in chickens infected by *S*. Typhimurium compared to values registered in animals challenged only with the pathogen [23]. Similarly, *L. acidophilus*, in combination with an aqueous extract from *Thymus vulgaris*, was capable of augmenting villus surface area in the jejunum of *S*. Enteritidis-challenged chickens [65]. Overall, the present outcomes corroborate previous reports showing that *Lactobacillus* administration alleviates intestinal impairments caused by *Salmonella* infections [17,66], which may have beneficial repercussions on nutrient absorption during later critical stages of growth.

Goblet cells make up part of the luminal surface and produce large amounts of a glycoprotein called Mucin 2 [67,68]. Intestinal gel-forming mucins procedure a highly protective viscous layer, which is known to play an important role during infection by pathogenic microorganisms [69]. *S*. Infantis augmented cell counts in the duodenum but did not alter this parameter in the jejunum and ileum. On the other hand, broiler chickens infected with *S*. Enteritidis showed a reduced number of goblet cells in the jejunum compared to control conditions [70]. Similar results were reported in chicks challenged with *S*. Pullorum, in which an important loss of goblet cells was observed in jejunal sections [22]. Treatment with probiotics diminished the negative effects triggered by the aforementioned serovars [22,70]. Indeed, exposure to *L. reuteri* induced epithelial cell proliferation and goblet cell differentiation [71]. Here, we have shown that administration of *L. fermentum* not only augmented the number of goblet cells in the duodenum and jejunum compared to the control group but also maintained such conditions despite infection by *S*. Infantis. Indeed, in chickens exposed to both bacteria, goblet cell count was observed to be the largest, demonstrating the utility of this probiotic to avoid potential barrier dysfunction caused by *Salmonella* colonization. Bacteria are known for their associated molecular patterns and secreted products that lead to the activation of the host receptors on epithelial and immune cells; this activation prompts goblet cell differentiation via cellular signaling or cytokine secretion [27]. Despite detecting a small increase in the number of goblet cells in the ileum, no changes were observed concerning transcriptional abundance of *muc-2*. Similarly, in *S*. Typhimurium-challenged mice, it has been observed that probiotic inoculation did not modify *muc-2* levels in colon samples [72]. Relative expression of *muc-2* has been observed to increase after *L. reuteri* administration in young chicks compared to control conditions, although in probiotic treated animals, goblet cell count almost doubled that of untreated birds [71].

In broiler chickens, *L. fermentum* application has been shown to increase plasma immunoglobulin (IgA and IgM) levels [73]. Furthermore, inoculation with this species augmented the percentage of IgA and IgM cells in the cecal lamina propria of chickens challenged with *C. coli* [26]. Here, we demonstrated that *L. fermentum* is also able to modulate IgM serum levels in chickens infected with *S*. Infantis, although no changes were observed regarding IgA production. Serum IgM is the first antibody to act after infection and contributes with pathogen clearance [74]. Higher levels of serum IgM, compared to control conditions, have been reported after simultaneous exposure to *L. plantarum* and *S*. Enteritidis in mice [74]. No effects on immunoglobulin concentration were registered in chickens infected with *S*. Infantis; indeed, this serovar is not considered as invasive as others [19].

IgA plays a key role in protecting the mucosal surface by neutralizing or preventing bacteria, viruses, or toxins from binding the intestinal epithelium [75]. IgA levels were not altered in response to any treatment. In line with our results, previous studies revealed that administration of a mixture of probiotics, including *L. reuteri* and *L. salivarius*, did not change plasma IgA levels [76,77]. In contrast, infection with *S*. Pullorum triggered production of plasma IgA, IgM, and IgG; such abundance was modulated by dietary administration of *L. casei* [22]. Certainly, administration of certain probiotics, including *L. fermentum*, might enhance the immune capacity of birds for coping with infections, especially with the host ability to handle long-term *Salmonella* colonization [78].

## 5. Conclusions

*S*. Infantis is associated with human salmonellosis and has been commonly reported in poultry-derived products, so it is considered a threat to public health. However, information is scarce concerning its effects on intestinal morphometry and immunoglobulin serum levels in broiler chickens. In this investigation, we showed that *S*. Infantis reduced villus-height-to-crypt-depth ratios in the duodenum, jejunum, and ileum compared to control conditions. The number of goblet cells was not altered, and in ileal sections, *muc-2* expression remained similar to those of the control. Similarly, the abundance of serum IgM and IgA was not modified by infection. Administration of *L. fermentum* not only ameliorated VH:CD ratios but also increased surface area and goblet cell count compared to control animals; this effect was also observed in *S*. Infantis-challenged birds. IgM serum levels were augmented in response to colonization by the probiotic in challenged chickens. Treatment with probiotic *Lactobacilli* elicited positive effects on the intestine and immunoglobulin serum levels and also relieved the outcomes triggered by *S*. Infantis. Undoubtedly, *L. fermentum* appears convenient for developing novel probiotic/prebiotic treatments aimed at reducing unwanted infections.

## Figures and Tables

**Table 1:** Expression levels of ileal *muc-2* in probiotic and pathogen-treated broiler chickens.

Expression Levels (2^−Δ^Ct)				
Gene symbol	Control	*L. fermentum*	*S.* Infantis	*L. fermentum* + *S.* Infantis
*muc-2*	0.107 ± 0.216	0.256 ± 0.613	0.159 ± 0.348	0.455 ± 1.262

Values represent means ± SD (*n* = 10). SD: standard deviation.

## Data Availability

Data supporting reported results can be found in Table S3: Experimental data.

## Supplementary Materials

The following supporting information can be downloaded at: https://www.mdpi.com/article/10.3390/microorganisms11020256/s1, Table S1: Experimental design; Table S2: COBB 500 Feed components and proximate composition of starter and grower diets; Table S3: Experimental data.
