# Supplementary material for: Effects of Lactobacillus fermentum Administration on Intestinal Morphometry and Antibody Serum Levels in Salmonella-Infantis-Challenged Chickens
Source: Microorganisms. 2023 Jan 19;11(2):256. doi: 10.3390/microorganisms11020256 (PMC9963312; doi:10.3390/microorganisms11020256)
Supplement: Supplementary file 1 [file microorganisms-11-00256-s001.zip › Table S1. Experimental design..pdf]

Table S1. Experimental design.

Day	**Control,** orally	**Lf** ***L. fermentum*** **treatment,** orally	**Si** ***S.*** **Infantis treatment,** orally	**LfSi Co-exposure treatment,** orally
1 – 3 d	Saline solution	10^9 CFU/0.2 mL (Probiotic)	Saline solution	10^9 CFU/0.2 mL (Probiotic)
4 d	Saline solution	10^9 CFU/0.2 mL (Probiotic)	10^7 CFU/0.1 mL (*S.* Infantis)	10^9 CFU/0.2 mL (Probiotic) + 10^7 CFU/0.1 mL (*S.* Infantis)
5 – 7 d (1 dpi – 3 dpi)	Saline solution	10^9 CFU/0.2 mL (Probiotic)	Saline solution	10^9 CFU/0.2 mL (Probiotic)
8 – 14 d (4 dpi – 10 dpi)	NT	NT	NT	NT
15 d (11 dpi) sample collection	10	10	10	10

NT, no treatment; dpi, days post-infection.
