# Supplementary material for: Effects of Lactobacillus fermentum Administration on Intestinal Morphometry and Antibody Serum Levels in Salmonella-Infantis-Challenged Chickens
Source: Microorganisms. 2023 Jan 19;11(2):256. doi: 10.3390/microorganisms11020256 (PMC9963312; doi:10.3390/microorganisms11020256)
Supplement: Supplementary file 1 [file microorganisms-11-00256-s001.zip › Table S2. COBB 500 Feed components and proximate composition of starter and grower diets.pdf]

Table S2. COBB 500 Feed components and proximate composition of starter (0–8 days) and grower (9–14 days) diets.

**Ingredients (%)**	**starter**	**grower**
Ground corn	52.99	57.23
Soybean meal	36.3	32.07
Calcium carbonate	1.52	1.49
Monocalcium phosphate	1.07	0.82
Sodium chloride	0.31	0.26
Crude (vegetal) Fat	7.4	7.71
Antimycotic	0.10	0.08
Mycotoxin Sequestrant	0.05	0.05
Antioxidant	0.02	0.02
Phytase	0.01	0.01
[1]Vitamin and mineral premix	0.23	0.26
**Nutrient specifications**		
[2]ME Kcal/kg diet	2975	3025
Digestible Lysine (%)	1.22	1.12
Digestible Methionine + Cysteine (%)	0.91	0.85
Digestible Methionine (%)	0.46	0.45
Digestible Threonine (%)	0.83	0.73
Digestible Valine (%)	0.89	0.85
Digestible Isoleucine (%)	0.77	0.72
Digestible Arginine (%)	1.28	1.18
Digestible Tryptophan (%)	0.20	0.18
Crude protein (%)	21.50	20.00
Ca (%)	0.90	0.84
Available P (%)	0.45	0.42
Na (%)	0.23	0.16
Cl (%)	0.22	0.19
K (%)	0.95	0.72
Choline (mg/kg)	500	400
Linoleic acid (%)	1.00	1.00

[1]Vitamin premix incorporated in each kg of basal diets: Vitamin A 10,000 IU; vitamin D3 5000 IU; vitamin E 80 IU (starter), 50 IU (grower); vitamin K 3 mg; vitamin B1 3 mg (starter), 2 mg (grower); vitamin B2 9 mg (starter), 8 mg (grower); vitamin B6 4 mg (starter), 3 mg (grower); vitamin B12 0.02 mg (starter), 0.015 mg (grower); Biotin 0.15 mg (starter), 0.12 mg (grower); Pantothenic acid 15 mg (starter), 12 mg (grower); Folic acid 2 mg. Mineral premix incorporated in each kg of basal diets: Mn, 100 mg; Zn, 100 mg; Fe, 40 mg, Cu, 15 mg, I, 1 mg, Se, 0.35 mg. [2]ME, metabolizable energy.
